# microRNA-139-5p exerts tumor suppressor function by targeting NOTCH1 in colorectal cancer

**DOI:** 10.1186/1476-4598-13-124

**Published:** 2014-05-26

**Authors:** Lijing Zhang, Yujuan Dong, Nana Zhu, Ho Tsoi, Zengren Zhao, Chung Wah Wu, Kunning Wang, Shu Zheng, Simon SM Ng, Francis KL Chan, Joseph JY Sung, Jun Yu

**Affiliations:** 1 Institute of Digestive Disease and Department of Medicine and Therapeutics, State Key laboratory of Digestive Disease, Prince of Wales Hospital, Li Ka Shing Institute of Health Sciences, Shenzhen Research Institute, The Chinese University of Hong Kong, Sha Tin, Hong Kong, China; 2 Departments of Surgery, First affiliated Hospital, Hebei Medical University, Shijiazhuang, China; 3 Department of Surgery, The Chinese University of Hong Kong, Sha Tin, Hong Kong, China; 4 The Key Laboratory of Cancer Prevention and Intervention of China National Ministry of Education and Cancer Institute, The Second Affiliated Hospital, Zhejiang University School of Medicine, Hangzhou, China

**Keywords:** miR-139-5p, Colorectal cancer, NOTCH1, Tumor suppressor

## Abstract

**Background:**

miR-139-5p was identified to be significantly down-regulated in colon tumor tissues by miRNA array. We aimed to clarify its biological function, molecular mechanisms and direct target gene in colorectal cancer (CRC).

**Methods:**

The biological function of miR-139-5p was examined by cell growth, cell cycle and apoptosis analysis *in vitro* and *in vivo*. miR-139-5p target gene and signaling pathway was identified by luciferase activity assay and western blot.

**Results:**

miR-139-5p was significantly down-regulated in primary tumor tissues (*P* < 0.0001). Ectopic expression of miR-139-5p in colon cancer cell lines significantly suppressed cell growth as evidenced by cell viability assay (*P* < 0.001) and colony formation assay (*P* < 0.01) and in xenograft tumor growth in nude mice (*P* < 0.01). miR-139-5p induced apoptosis (*P* < 0.01), concomitantly with up-regulation of key apoptosis genes including cleaved caspase-8, caspase-3, caspase-7 and PARP. miR-139-5p also caused cell cycle arrest in G0/G1 phase (*P* < 0.01), with upregulation of key G0/G1 phase regulators p21^Cip1/Waf1^ and p27^Kip1^. Moreover, miR-139-5p inhibited cellular migration (*P* < 0.001) and invasiveness (*P* < 0.001) through the inhibition of matrix metalloproteinases (MMP)7 and MMP9. Oncogene NOTCH1 was revealed to be a putative target of miR-139-5p, which was inversely correlated with miR-139-5p expression (r = -0.3862, *P* = 0.0002).

**Conclusions:**

miR-139-5p plays a pivotal role in colon cancer through inhibiting cell proliferation, metastasis, and promoting apoptosis and cell cycle arrest by targeting oncogenic NOTCH1.

## Background

Colorectal cancer (CRC) is the third most common malignancy worldwide and the second leading cause of tumor-related death in Western countries [1]. According to International Agency for Research on Cancer, approximately 1.24 million new cases of CRC were reported worldwide in 2008 [1]. CRC incidence rates are rapidly rising in Asian countries [2], which were formerly considered as low-risk area. So there is a growing need for underling the molecular pathogenesis of CRC. Over the last decade, microRNAs (miRNAs) have emerged as key players in carcinogenesis. Aberrant expression of miRNAs has been demonstrated to play a critical role in the initiation and progression of several human cancers through post-transcriptional regulation of gene expression [3-5].

It has been shown that miRNAs play an important role in the development of CRC. miRNAs in CRC exhibit oncogenic or tumor suppressive role by directly regulating oncogenes or tumor-suppressor genes [6,7]. Our previous work on miRNA microarray analysis revealed that miR-139-5p was downregulated in colon cancers (0.10 ~ 0.15-fold of the non-tumor miR-139-5p) [8,9]. However, the functional role and mechanistic action of miR-139-5p in CRC remain largely unclear. In this study, we aimed to determine its biological function, molecular basis and target gene in CRC.

## Results

### miR-139-5p is frequently downregulated in primary CRC and colon cancer cell lines

**Figure 1:**
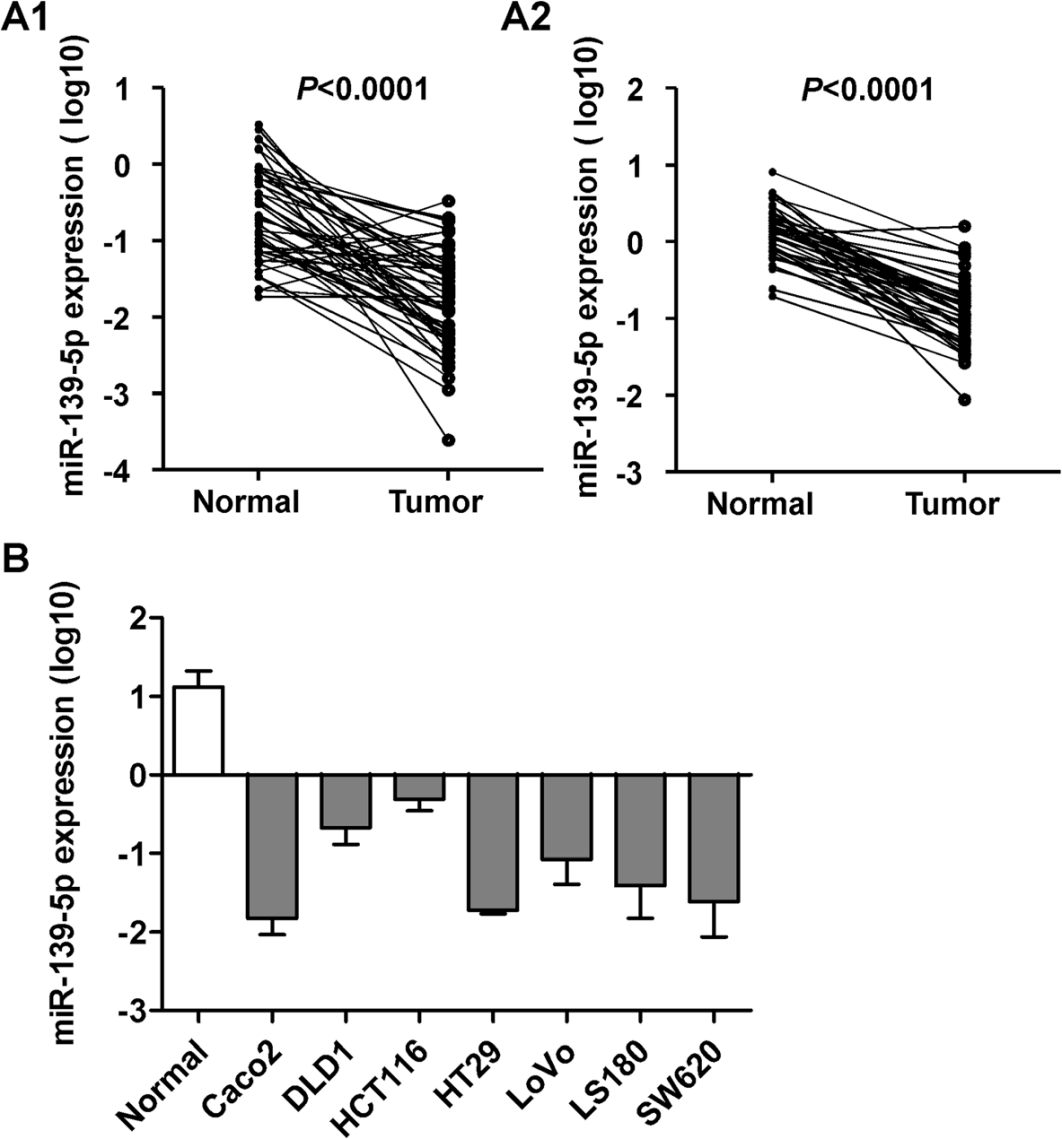
**miR-139-5p was down-regulated in primary colorectal cancer tissues and colon cancer cell lines. (A)** Levels of miR-139-5p in primary colorectal tumors compared with their adjacent normal tissues (A1, Cohort 1, n = 45; A2, Cohort 2, n = 50). **(B)** The expression level of miR-139-5p in seven colon cancer cell lines and normal colorectal tissues (n = 9) by qRT-PCR.

We previously performed miRNA arrays based on five CRC patients and two advanced adenoma patients [8,9]. The miRNA array results showed that miR-139-5p was significantly down-regulated in both CRC and advanced adenoma compared with their adjacent normal tissues. The Sanger database shows that miR-139-5p shares the same precursor with miR-139-3p. However, miR-139-3p was undetectable in CRC and the adjacent non-tumor tissues. Therefore, our study focused on the miR-139-5p. The downregulation of miR-139-5p in CRC was further verified in two independent cohorts of colon cancer patients (cohort 1, n = 45; and cohort 2, n = 50, Additional file 1: Table S1) and 7 colon cancer cell lines. Consistent with the findings from miRNA array, there was a significant decrease in the expression level of miR-139-5p in both cohorts of CRC tumors when compared with their matched non-tumorous tissues (*P* < 0.0001 in both cohorts) (Figure 1A). We had performed qRT-PCR to determine the level of miR-139-5p. Among the paired samples, 89% (40/45) of cohort 1 and 98% (49/50) of cohort 2 CRC samples showed lower miR-139-5p levels than in the adjacent normal tissue. In addition, we examined miR-139-5p expression levels in seven colon cancer cell lines (Caco2, DLD1, HCT116, HT29, LoVo, LS180 and SW620). We observed that miR-139-5p was downregulated in all seven colon cancer cell lines relative to nine normal colon tissues (*P* < 0.0001) (Figure 1B). Moreover, miR-139-5p expressions were restored in 5 CRC cell lines upon administration of DNA methylation inhibitor 5-aza-2’-deoxycytidine (Additional file 2: Figure S1A), which suggests that epigenetic silencing may at least contributed to the down-regulation of miR-139-5p. The correlation of miR-139-5p expression with clinicopathological features of 44 CRC patients was investigated. Based on univariate analysis of this cohort, miR-139-5p was not associated with clinicopathological features including gender, TNM stage, tumor size and recurrence. Kaplan-Meier survival analysis showed that miR-139-5p expression level was not associated with disease-free survival and overall survival of CRC patients (Additional file 3: Table S2 and Additional file 4: Figure S2).

### miR-139-5p suppresses colon cancer cell growth *in vitro* and *in vivo*

**Figure 2:**
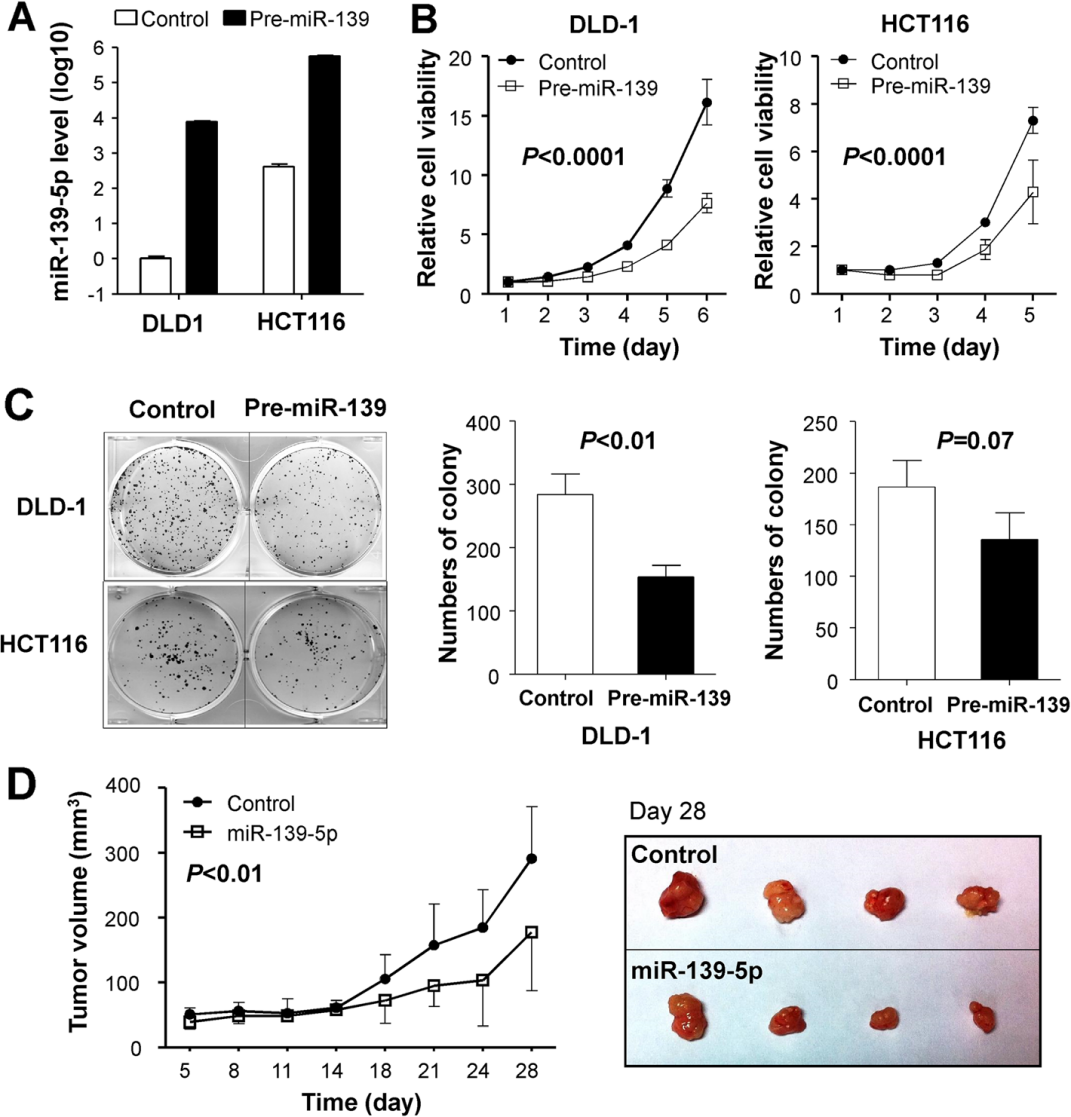
**miR-139-5p inhibited colon cancer cell growth *in vitro *and *in vivo*. (A)** Ectopic expression of miR-139-5p in colon cancer cell lines DLD1 and HCT116 were evidence by qRT-PCR after transfection of pre-miR-139. **(B)** miR-139-5p significantly inhibited DLD1 and HCT116 cell viability. Cell growth rates were detected by MTS assay. **(C)** miR-139-5p inhibited DLD1 and HCT116 cells colony formation (left panel). Quantitative analysis of DLD1 and HCT116 colony numbers (n = 3, mean ± SD) (right panel). **(D)** miR-139-5p inhibited colon cancer cell growth *in vivo*. Tumor growth curve of pre-miR-139-5p and control transfected HCT116 cells in nude mice. Data are mean ± SD (n = 4/group) (left panel). Images of the tumors induced by pre-miR-139-5p or control were shown (right panel).

The frequent downregulation of miR-139-5p in both primary CRC tumor tissues and colon cancer cell lines indicates that miR-139-5p might play a role in CRC tumorigenesis. To explore the potential tumor suppressive effect of miR-139-5p, miR-139-5p precursor (pre-miR-139) was transfected into colon cancer cell lines (DLD1, HCT116 and SW1116). We performed real-time PCR to check the expression level of miR-139-5p and miR-139-3p after transfected with pre-miR-139 mimics. We observed an increase of miR-139-5p post-transfection, while miR-139-3p showed no significantly changes (Additional file 2: Figure S1B). Our results demonstrated that pre-miR-139 mimic conferred to up-regulation of miR-139-5p, but not miR-139-3p. Ectopic expression of miR-139-5p in DLD1 and HCT116 cells caused a significant decrease in cell viability (*P* < 0.0001 in both cell lines) (Figure 2A and B). The inhibitory effect of miR-139-5p on cancer cell growth was further confirmed by colony formation assay. The colonies formed in pre-miR-139-transfected cells were significantly less than in control-transfected cells in DLD1 and a borderline statistically significant was observed in HCT116 as compared with control (*P* = 0.07) (Figure 2C). To further confirm the growth inhibitory effect of miR-139-5p on colon cancer cells, a xenograft tumor growth assay was performed. The subcutaneous tumor growth curve of HCT116 stably expressed miR-139-5p or control *in vivo* was shown in Figure 2D. The tumor volume was significantly lower in miR-139-5p nude mice as compared to the control mice (*P* < 0.01). These results provided further evidence that miR-139-5p plays a tumor suppressive role in colon cancer.

### miR-139-5p induces G0/G1 cell cycle arrest in colon cancer cells by induction of p53, p21^Cip1/Waf1^ and p27^Kip1^

**Figure 3:**
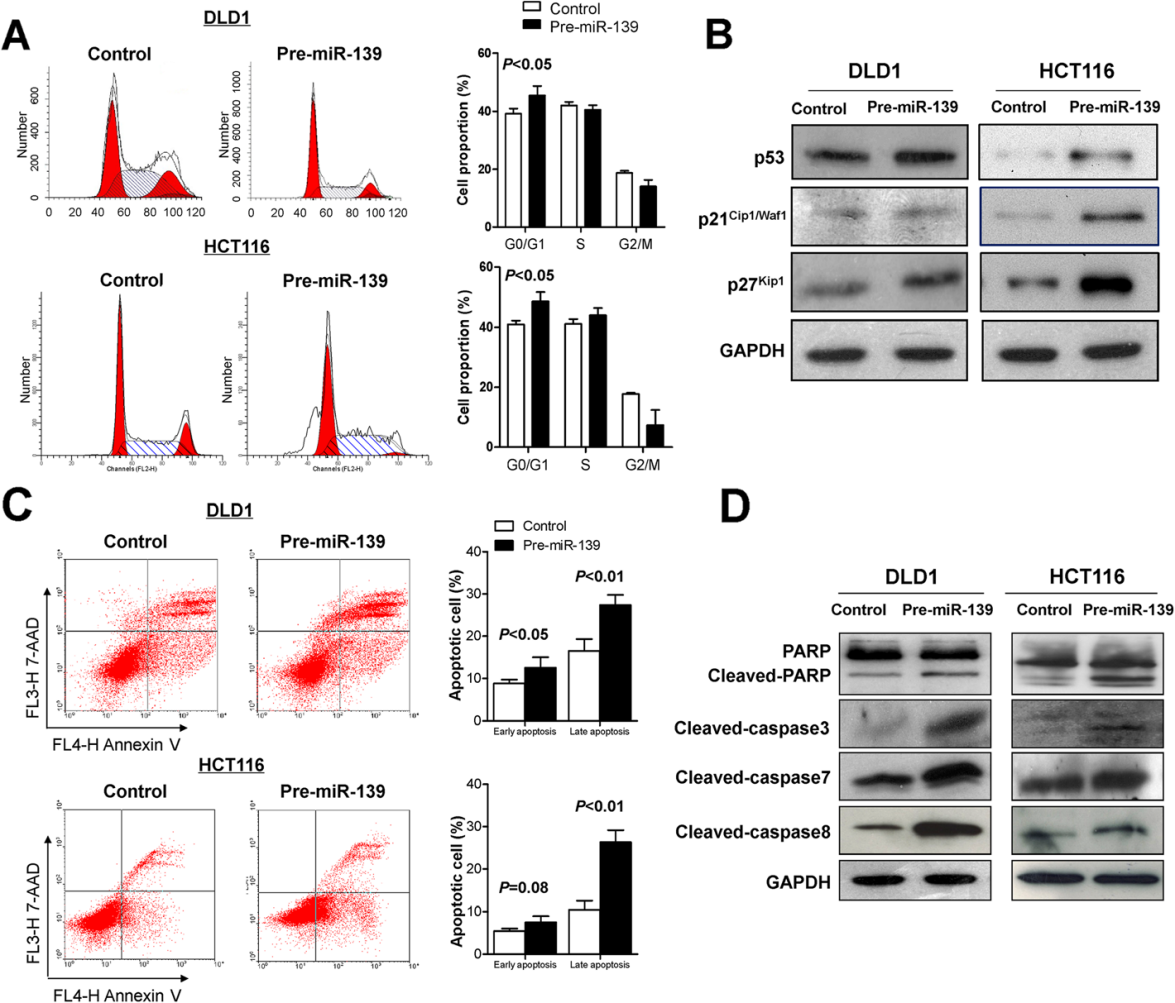
**miR-139-5p induced colon cancer cell apoptosis and cell cycle arrest at G0/G1 phase. (A)** miR-139-5p caused cell cycle arrest at G0/G1 phase in DLD1 and HCT116 cells (left panel). Quantitative analysis of G0/G1 proportion in DLD1 and HCT116, The experiments were repeated three times in triplicate. Data are mean ± SD. **(B)** G0/G1 phase cell cycle related protein expression including p21^Cip1/Waf1^, p27^Kip1^ and p53 were determined by western blot in DLD1 and HCT116 cells **(C)** miR-139-5p induced apoptosis in DLD1 and HCT116 cells. Cell apoptosis was examined by flow cytometry analysis of Annexin V-APC and 7-AAD double-staining. Data represented the average of the early and late apoptotic cells, respectively. **(D)** Apoptotic-related protein expression (PARP, caspase-3, 7, 8) were detected by western blot.

We investigated the effect of miR-139-5p on cell cycle distribution. Concomitant with this inhibition of cell proliferation, there was a significant increase in the number of cells accumulating in the G0/G1 phase in DLD1 and HCT116 (Figure 3A). p21^Cip1/Waf1^ and p27^Kip1^ are two important cyclin-Cdk inhibitors that negatively regulate G1 phase progression. Therefore, we determined the expression levels of these two cell cycle inhibitors in pre-miR-139-transfected. Ectopic expression of miR-139-5p induced p53 and p27^Kip1^ protein expression in both DLD1 and HCT116 cells (Figure 3B); however, upregulation of p21^Cip1/Waf1^ was only observed in HCT116 cells (Figure 3B). Thus, miR-139-5p suppressed cell proliferation by inducing a G0/G1 cell cycle arrest mediated by induction of p21^Cip1/Waf1^ and p27^Kip1^.

### miR-139-5p induces cell apoptosis by regulating caspase-dependent apoptosis pathway

In order to determine whether the decrease in colon cell growth by miR-139-5p was due to an induction of apoptosis, we evaluated the rate of cellular apoptosis using Annexin V-APC and 7-AAD staining by flow cytometry. As shown in Figure 3C, the number of both early and late apoptotic cells at 48–96 h post-transfection of pre-miR-139 in DLD1 and HCT116 cells was substantially increased as compared to control pre-miRNA transfected DLD1 and HCT116 cells. Induction of apoptosis was further confirmed by the expression of apoptosis-related proteins using western blot. As shown in Figure 3D, the protein levels of the active forms of caspase-3, caspase-7, caspase-8 were enhanced. To further confirm their activities in active form, the cleavage of nuclear poly (ADP-ribose) polymerase (PARP) was determined. As expected, the level of cleaved PARP was enhanced in the pre-miR-139-transfected cells compared to the control cells both in DLD1 and in HCT116 (Figure 3D). These results inferred that apoptosis concomitant with cell cycle arrest induced by miR-139-5p was contributing factors leading to the growth inhibition in miR-139-5p-expressing colon cancer cells.

### miR-139-5p suppresses CRC cell migration and invasiveness of colon cancer cell lines

**Figure 4:**
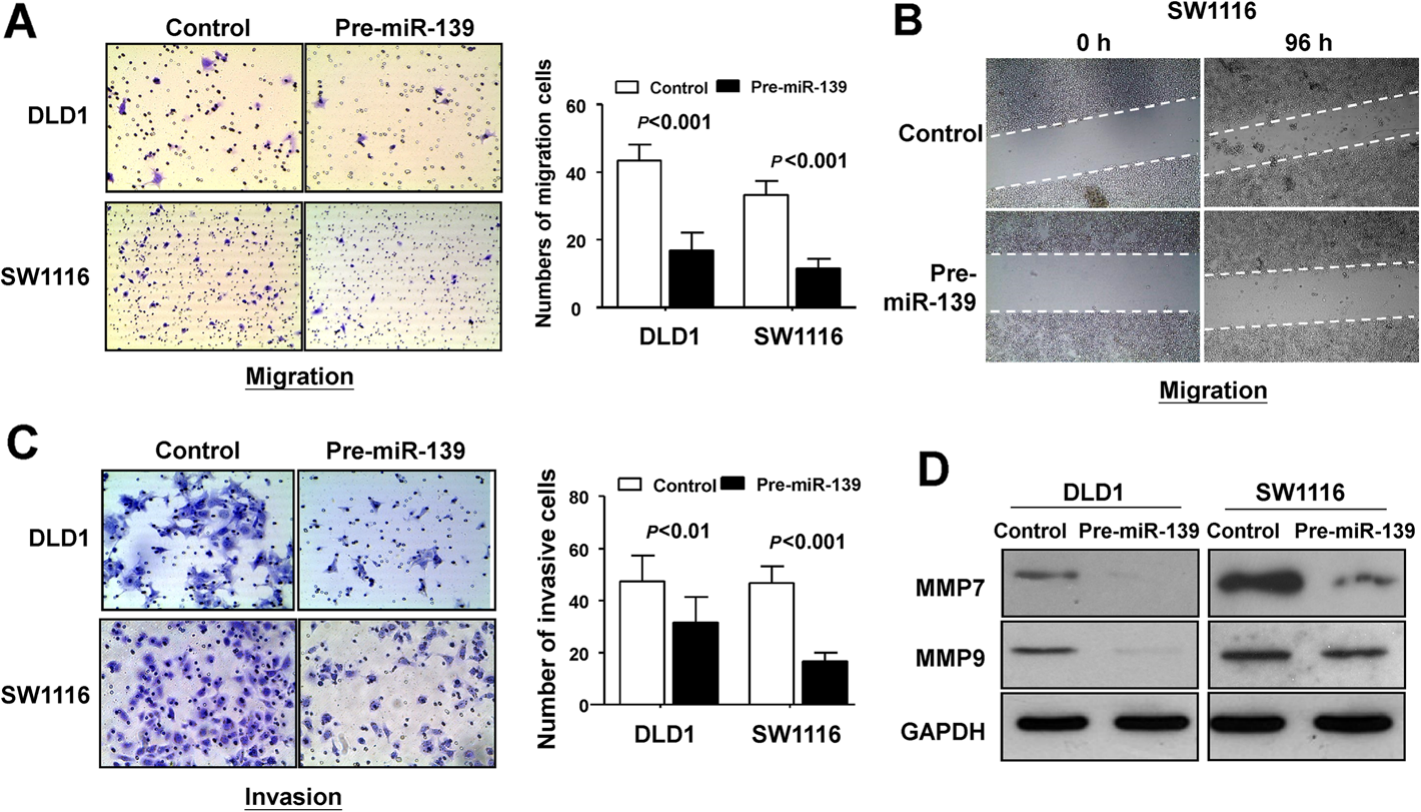
**miR-139-5p inhibited colon cancer cell migration and invasion. (A)** Re-expression of miR-139-5p in DLD1 and SW1116 cells significantly inhibited cell migration ability as determined by cell migration assay (left panel) Quantitative analysis of migrated cell in DLD1 and SW1116. The data are means ± SD. **(B)** The inhibition on cell migration by miR-139-5p was further confirmed by wound-healing assay. **(C)** Re-expression of miR-139-5p in DLD1 and SW1116 cells significantly suppressed cell invasive ability as determined by cell invasion assay. **(D)** miR-139-5p significantly downregulated MMP7 and MMP9 protein levels in both colon cancer cell lines.

We next tested whether miR-139-5p could alter migration and invasiveness of colon cancer cells *in vitro*. DLD1 and SW1116 cells were transfected with pre-miR-139 or control induction of miR-139-5p significantly decreased the migrated cells in DLD1 (*P* < 0.001) and in SW1116 (*P* < 0.001) compared to control cells (Figure 4A). Moreover, miR-139-5p markedly slowed cell migration scratchy “wound” at edges of SW1116 and HCT116 cells (Figure 4B and Additional file 5: Figure S3). The reduction in wound closure by miR-139-5p further confirmed its effect in suppressing cell migration.

To study the effect of miR-139-5p on the invasiveness of colon cancer, DLD1 and SW1116 cells were transfected with pre-miR-139 or Control miRNA using a Matrigel model (Figure 4C). *In vitro* invasively growing colon cancer cells were significantly impaired in DLD1 (*P* < 0.01) and in SW1116 (*P* < 0.01) when transfected with pre-miR-139 (Figure 4C).

The matrix metalloproteinases (MMPs) are family members of extracellular proteinases that regulate basic cellular processes including survival, migration and morphogenesis and degradation extracellular matrix during the cancer metastatic process [10,11]. Among the MMPs members, MMP7 and MMP9 cleave the extracellular domain of NOTCH1 and activate NOTCH1 downstream target gene expression [12,13]. Therefore, we examined the expression levels of these two proteinases and found that ectopic expression of miR-139-5p markedly suppressed protein expression of MMP7 and MMP9 (Figure 4D), concomitant with the inhibition of cell migration and invasion.

### miR-139-5p targets *NOTCH1* via binding to its 3’UTR

**Figure 5:**
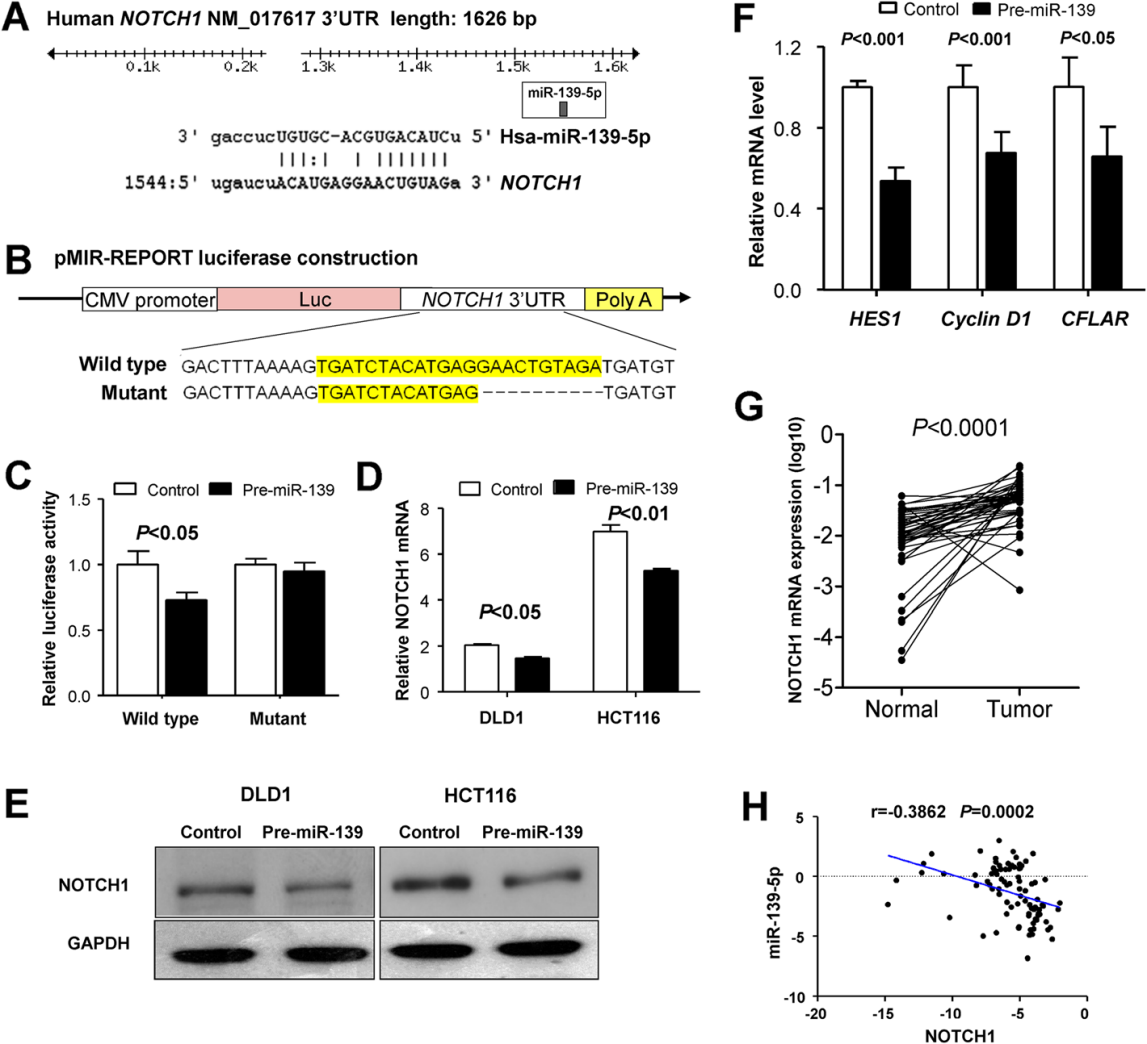
**NOTCH1 is a direct target of miR-139-5p in colon cancer. (A)** Human NOTCH1 3’UTR binding site for miR-139-5p. **(B)** The miR-139-5p wild type binding sequence or its mutated form was inserted into C-terminal of the luciferase gene to generate pMIR-NOTCH1-3’UTR or pMIR-NOTCH1-mut-3’UTR, respectively. **(C)** miR-139-5p targeted the wild-type but not the mutant 3’UTR of NOTCH1. The data are means ± SD. **(D)** Ectopic expression of miR-139-5p downregulated NOTCH1 mRNA expression in DLD1 and HCT116 cells as determined by quantitative RT-PCR. **(E)** miR-139-5p decreased NOTCH1 protein level in DLD1 and HCT116 by western blot. **(F)** miR-139-5p significantly suppressed NOTCH1 downstream effectors including *hairy and enhancer of split-1* (*HES1*), *cyclin D1* and *FADD-like apoptosis regulator* (*CFLAR*) transcription. **(G)** Expression of NOTCH1 is significantly upregulated in primary colorectal tumors compared with their adjacent normal tissues (n = 45). **(H)** Scatter plots showing the inverse association between miR-139-5p level and *NOTCH1* mRNA expression.

We searched for the direct target of miR-139-5p based on the following criteria: the target should have oncogenic property and regulate the cell migration and invasion. Among the top 100 targets of miR-139-5p predicted by 2 bioinformatics algorithms (TargetScan and miRanda), NOTCH1 fit our criteria. The 3' untranslated region (3'UTR) of *NOTCH1* contains a conserved binding site for miR-139-5p (Figure 5A). To test the specific regulation through the predicted binding sites, we constructed a reporter vector which consists of the luciferase coding sequence followed by the 3’UTR of *NOTCH1* (Luc-NOTCH1-3’UTR) (Figure 5B). Wild type (Luc-NOTCH1-3’UTR) or mutated sequence (Luc-NOTCH1-mut 3’UTR) within the putative binding sites was cloned into the pMIR-REPORT vector (Figure 5A and B). Co-transfection experiments in HCT116 cells showed that miR-139-5p significantly decreased the luciferase activity of Luc-NOTCH1-3’UTR (P < 0.05), but this was not observed in Luc-NOTCH1-mut 3’UTR (Figure 5C). Our data thus demonstrated that *NOTCH1* was a direct target of miR-139-5p.

To further confirm that miR-139-5p targets NOTCH1, pre-miR-139 or control was transfected into DLD1 and HCT116 cells. Transfection of pre-miR-139 resulted in significant reduction of NOTCH1 mRNA and protein expression by qRT-PCR (Figure 5D) and by western blot (Figure 5E), respectively. Consistent with this finding, ectopic expression of miR-139-5p significantly suppressed *NOTCH1* downstream effectors, including *hairy and enhancer of split-1* (*HES1*) (*P* < 0.001), *Cyclin D1* (*P* < 0.001) and *FADD-like apoptosis regulator* (*CFLAR*) (*P* < 0.05) transcription (Figure 5F). Collectively, these results showed that mir-139-5p could regulate NOTCH1 and its mediated signal transduction.

### NOTCH1 is upregulated in primary CRC tumors and inverse correlated with miR-139-5p expression

To assess the importance of NOTCH1 in primary CRC, we compared the level of NOTCH1 expression in 45 paired tumor and adjacent normal tissues. The expression of the *NOTCH1* mRNA was significantly increased in CRC tumors compared to the adjacent normal tissues (*P* < 0.0001; Figure 5G). NOTCH1 was overexpressed in 91% (41/45) of tumors compared with their normal counterparts.

We evaluated the correlation between *NOTCH1* mRNA and miR-139-5p expression in 45 pairs of primary CRC. Expression of *NOTCH1* mRNA and miR-139-5p exhibited a significant inverse correlation as calculated by Pearson correlation (r = -0.3862, *P* = 0.0002) (Figure 5H). This result further supported that miR-139-5p targets to NOTCH1.

We investigated the effect of NOTCH1 on cell growth by NOTCH1 knockdown experiment. Knock-down of NOTCH1 was evidenced by markedly decrease of NOTCH1 protein expression (Additional file 6: Figure S4A). Knockdown of NOTCH1 caused a significant reduction in cell viability and an increased G0/G1 phase in HCT116 (*P* < 0.0001, Additional file 6: Figure S4B and S4C), indicating the potential oncogenic function of NOTCH1 in colon cancer.

## Discussion

miR-139-5p is down-regulated in inflammatory bowel disease-associated neoplastic transformation, primary CRC and the metastasis site [14-18]. Our data also indicated that miR-139-5p showed a reduced expression in advanced adenoma, suggesting the dysregulation of miR-139-5p is an early event of colorectal tumorigenesis. Further work on two cohorts of CRC patients evaluated by qRT-PCR verified that miR-139-5p was indeed dramatically downregulated in tumor samples compared to the adjacent normal tissues (*P* < 0.0001 in both cohorts) (Figure 1). In addition to CRC, down-regulation of miR-139-5p has been reported by miRNA profile studies on gastric cancer [19], endometrial serous adenocarcinoma [20] and HCC [21]. Epigenetic silencing of miRNAs with tumor suppressor features is a common hallmark of human tumors. Human miR-139-5p is embedded in the intronic region of PDE2A gene on chromosome 11q13.4. Most recently it was documented that miR-139 was silenced with its host gene PDE2A through histone methylation in lung cancer [22]. EZH2 was also reported to silence miR-139-5p through H3K27 methylation in human hepatocellular carcinoma [23]. In gastric cancer, HER2 cooperates with CD44 and down-regulates miR-139 via histone H3K9 deacetylation in the miR-139 promoter region [24]. In colorectal cancer, we have found that miR-139-5p expression is restored in CRC cell lines upon administration of DNA methylation inhibitor 5-aza-2’-deoxycytidine (Additional file 2: Figure S1A). This suggests that epigenetics regulation could contribute to the down-regulation of miR-139-5p. Except for the epigenetics regulation, Schepeler and colleagues reported that WNT pathway suppressed the expression of miR-139-5p in CRC. Abrogation of the WNT pathway induced the expression of miR-139-5p but not miR-139-3p in DLD1 cell [18]. Collectively, silencing or downregulation of miR-139-5p can be mediated by different mechanisms in human cancer.

We therefore characterized the putative tumor suppressive function of miR-139-5p in human colon cancer cell lines. We found that the restoration of miR-139-5p in the colon cancer cell lines DLD1 and HCT116 significantly inhibited cell proliferation as evidenced by cell viability and colony formation assays (Figure 2). In nude mice, colon cancer cells over-expressing miR-139-5p displayed a significantly lower growth rate than the control cancer cells (Figure 2D). Furthermore, we performed FACS to evaluate the effects of miR-139-5p on the cell cycle in colon cancer cells. We revealed a concomitant increase of cells in G0/G1, which lead to inhibition of cell proliferation (Figure 3A). Previous study suggested that miR-139-5p did not affect the proliferation phenotype and DNA profile of breast cancer cell MDA-MB-231 based on Doxycycline-induced plasmid expression [25]. However, miR-139-5p shows an anti-proliferative effect in colorectal cancer. This discrepancy may be attributed to a tissue specific function of miR-139-5p signaling in CRC compared with other solid tumors. On the basis of the immunoblot analysis of negative cell-cycle regulators, G0/G1 arrest by miR-139-5p was most likely associated with the induction of p53, p21^Cip1/Waf1^ and p27^Kip1^ (Figure 3B). It is well known that p53 acts as a putative mediator to induce cell cycle arrest and to allow either DNA repair or apoptosis through transcriptional upregulation of the cyclin dependent kinase (CDK) inhibitor p21^Cip1/Waf1^, an active inhibitor of CDKs [26]. p21^Cip1/Waf1^ is a direct regulator of the cell cycle, inducing growth arrest in G1-phase of the cell cycle by binding to and thus inhibiting the activity of cyclinD-CDK2/4 complexes [27]. Increased protein expression of p21^Cip1/Waf1^ prevents cyclinD-CDK2/4 mediated phosphorylation of retinoblastoma protein (pRb), thus, inhibiting E2F transcriptional activity and cell cycle progression to S-phase [28]. p27^Kip1^ is a potent inhibitor of cyclin D/CDK4 and cyclin E/CDK2 activities. The role of p27^Kip1^ as a major player in G1 arrest has been well accepted [29]. In this regard, miR-139-5p mediated G1 cell cycle arrest in colon cancer may be partly contributed by a mechanism involving up-regulation of protein expression of p21^Cip1/Waf1^ and p27^Kip1^.

In the present work, we showed that, in addition to inhibition of cell proliferation, the growth inhibitory effect of miR-139-5p was also related to induction of apoptosis (Figure 3C). We observed that induction of miR-139-5p mediated apoptosis occurs by the modulation of extrinsic apoptosis pathway. Apoptosis induced by extrinsic pathways has been considered to be an important antitumor mechanism [30-32]. After transfection with miR-139-5p, the protein expression of the downstream active apoptosis executors caspase-8, caspase-7, caspase-3 was upregulated and enhanced level of cleaved PARP indicated caspase 3 was functionally active (Figure 3D). The extrinsic apoptosis pathway is initiated by the binding of extracellular death ligands such as TNFα to transmembrane death receptors. Engagement of death receptors with their cognate ligands provokes the recruitment of adaptor proteins such as Fas-associated death domain protein, which in turn recruits and aggregates several molecules of caspase-8, thereby promoting its auto-processing and activation [33]. Activation of caspase-8 processes other effector caspase members, including caspase-3 and caspase-7 to initiate a caspase cascade. These effectors further initiate the proteolytic cleavage of the nuclear enzyme PARP, causing loss of DNA repair, cellular disassembly and finally apoptosis.

*In vitro* assays showed that re-expression of miR-139-5p inhibited the cell migration and invasive capabilities (Figure 4 and Additional file 5: Figure S3). The reduced spreading effect and cell motility caused by miR-139-5p in colon cancer cells was revealed to be associated with the inhibition of the protein expression of cell migration and invasion molecules MMP7 and MMP9 (Figure 4D). MMP7 is an established instigator of aggressive behavior in a number of cancer types including CRC [34,35]. MMP9 has been identified as a critical component for priming of the pre-metastatic niche [36]. Thus, down-regulation of MMP7 and MMP9 expression by miR-139-5p contributed to dampened cell spreading and invasion ability. In keeping with our finding, a recently published study also suggested that a plasmid-based stable expression of miR-139-5p in HCT116 cells significantly suppressed cell migration and invasion [17].

Having shown the crucial role of miR-139-5p in suppressing CRC development, we sought for the possible gene effectors participating in its function. Of note, a single miRNA can regulate a multitude of target genes concomitantly; for instance, it has been reported that miR-139-5p suppresses progression of liver cancer by down-regulating Rho-kinase 2 [21]; and miR-139-5p could repress the activity of RAP1B [37] and IGF-IR [17] in colon cancer. Among the miRNAs predicted to target genes, we found that *NOTCH1* acts as a critical effector of miR-139-5p. We showed that miR-139-5p was able to significantly repress the luciferase activity of Luc-NOTCH1-3’UTR by targeting the 3’UTR of NOTCH1 mRNA (Figure 5A-C). We found that c-JUN also contained evolutionarily conserved binding site for miR-139-5p based on the in silicon search. However, miR-139-5p showed no effect on the wild type c-JUN-3’UTR or the mutant c-JUN-3’UTR reporter activity (Additional file 7: Figure S5). Therefore we focused on NOTCH1 for further analysis. We also successfully verified that downstream targets of NOTCH1 were negatively regulated by miR-139-5p including *HES1*, *cyclin D1* and *CFLAR* (Figure 5F), further reaffirming that miR-139-5p regulated NOTCH1 signal transduction by controlling the expression level of *NOTCH1*.

NOTCH signaling pathway consists of three components: the NOTCH ligands (JAG1, JAG2, DLL1, DLL3, DLL4), NOTCH receptors 1–4, and downstream target genes. Aberrantly activated NOTCH signaling has been observed during the carcinogenesis of several human cancers [38]. *NOTCH1* which is prominently expressed by epithelial cells of the crypts promotes tumor growth by enhancing the G1-S transition of the cell cycle and by increasing cell migration and invasion under pathological conditions [39-41]. We observed that the NOTCH1 mRNA expression was inversely correlated with miR-139-5p expression in CRC patients (r = -0.3862, *P* = 0.0002) (Figure 5H), suggesting miR-139-5p potentially inhibits NOTCH1 mRNA expression. In addition, other miRNAs (miR-34a, miR-144) were also reported to suppress NOTCH1 in CRC [42,43]. Subsequent functional validation revealed the decreased cell proliferation when NOTCH1 was downregulated in colon cancer cells (*P* < 0.0001) (Additional file 6: Figure S4B). In agreement with our results, it has been shown that suppression of NOTCH1 inhibits tumor growth in human HCC [44] and in osteosarcoma [45] by regulating cell proliferation and cycle. However, in the Kaplan-Meier survival analysis, there is no association between miR-139-5p or NOTCH1 mRNA and patient disease-free survival and overall survival in colorectal patients of our cohort (Additional file 4: Figure S2 and Additional file 8: Table S3).

Recent studies have reported that miR-139-5p was a potential prognostic marker for renal cell carcinoma and endometrial serous adenocarcinoma [46,20]. The clinical value of miR-139-5p in CRC is still controversial. It was reported that higher level of miR-139-5p was associated with more aggressive disease of colorectal cancer [16], whereas a recent study suggested that decreased miR-139-5p was associated with CRC disease progression and metastasis based on a cohort of 34 CRC patients [17]. In our study, miR-139-5p was not found to be associated with clinicopathological features including survival of CRC patients. The clinical value of miR-139-5p needs future investigations on larger cohorts of CRC samples.

## Conclusions

In conclusion, we have identified that miR-139-5p was frequently under-expressed in CRC. miR-139-5p inhibits cell growth both *in vitro* and *in vivo*. The mechanisms by which miR-139-5p acts as a tumor suppressor in CRC involve inhibition of cell proliferation and metastasis, and induction of G0/G1 arrest and apoptosis through repression of oncogenic NOTCH1.

## Methods

### Tumor cell lines

Eight colon cancer cell lines (Caco2, DLD1, HCT116, HT29, LoVo, LS180, SW620 and SW1116) were obtained from the American Type Culture Collection (ATCC, Manassas, VA). Cells were cultured in DMEM (Gibco BRL, Rockville, MD) and McCoy’s 5A modified (Invitrogen; Life Technologies, Carlsbad, CA) medium supplemented with 10% fetal bovine serum (FBS, HyClone, Logan, UT) and incubated in 5% CO_2_ at 37°C.

### Primary tumor and adjacent non-tumorous tissue samples

Two cohorts of totally 95 histologically confirmed sporadic CRC patients diagnosed in the Prince of Wales Hospital (between November 1999, and January 2011) were included in this study (Additional file 1: Table S1). In addition, nine biopsies of normal colon mucosa from healthy controls during colonoscopy were obtained in the Prince of Wales Hospital. All of the patients were given written informed consent according to the Helsinki declaration and the study protocol was approved by the Clinical Research Ethics Committee of the Chinese University of Hong Kong.

### Real-time quantitative PCR for miRNA expression analyses

Total RNA was extracted from cell pellets and tissue samples using miRNeasy Mini Kit (Qiagen, Hilden, Germany). Quantitative reverse transcription (qRT)-PCR of miR-139-5p was performed using the TaqMan miRNA reverse transcription kit (Applied Biosystems; Life Technologies) and the TaqMan human miRNA assay kit (assay ID: miR-139-5p: 002289, and RNU6B: 001093) [8]. The comparative Ct method was used to calculate the relative abundance of miRNA compared with RNU6B expression (Fold difference relative to RNU6B).

### miRNA transfection and RNA interference

Human miR-139-5p precursor (pre-miR-139, MC11749) and the miRNA Mimic Negative Control (control) were purchased from Ambion (Ambion Life Technologies, Austin, TX) and then transiently. Cell was seeded in 24-well plate at 60% confluence and transfected with 15 pmole miRNA per well using Lipofectamine 2000 (Invitrogen). siRNAs against human *NOTCH1* (Santa Cruz Biotechnology, Santa Cruz, CA) were delivered into cell using Lipofectamine 2000. Cells transfected with miRNA or siRNA were harvested 12 h to 48 h post-transfection.

### Cell viability assay

After 24 h of transfection of miRNA/siRNA, the cells were digested and re-seeded in 96-well plates (1.5 × 10^3^ per well) for cell viability assay using MTS (Promega, Madison, WI). 20 uL of reaction solution was added to cultured cells in 100 uL culture medium and incubated at 37°C for 1.5 h. The optical density was measured at a wavelength of 492 nm. The cell viability assay was carried out in four wells for three independent experiments.

### Colony formation assay

HCT116 and DLD1 cells (5 × 10^4^/well) were plated in a 24-well plate and transfected with pre-miR-139 or control RNA. After 24 h, the cells collected and seeded (1 000–1 500/well) in a fresh 6-well plate for 10 days. Surviving colonies (>50 cells per colony) were counted after fixed with methanol/acetone (1:1) and stained with 5% Gentian Violet (ICM Pharma, Singapore, Singapore). The experiment was carried out in triplicate wells for three times.

### Cell cycle analysis and apoptosis assay

Cells used for cell cycle analysis were plated 24 h prior to transfection with miRNAs in 24-well plate using Lipofectamine 2000 per manufacturer's instructions. Cells were trypsinized and then collected by centrifugation at 2,000 rpm for 6 min. Cell pellets were rinsed once with phosphate-buffered saline (PBS) and fixed in 70% (v/v) ice-cold ethanol for at least 16 h at -20°C. Cells were resuspended and stained with 50 μg/mL propidium iodide (BD Biosciences, Erembodegem, Belgium) containing RNase A for 30 min at 37°C in the dark. Apoptosis was determined by dual staining with Annexin V-Allophycocyanin (APC) and 7-Aminoactinomycin D (7-AAD) (BD Biosciences, Erembodegem, Belgium). Briefly, cells were collected 48 h after transfected with pre-miR-139 or control, and stained with Annexin V-APC and 7-AAD according to the manufacturer’s instruction. The combination of Annexin V-APC and 7-AAD staining distinguished early apoptotic cells (Annexin V+, 7-AAD-) and late apoptotic cells (Annexin V+, 7-AAD+). The cells were sorted by FACS-Calibur System (BD Biosciences) and cell-cycle profiles and apoptosis were analyzed by ModFit 3.0 software (BD Biosciences). The assay was carried out in triplicate for three times.

### Cell migration assays and wound healing assay

To measure cell migration, 8-mm pore size-culture inserts (Transwell; Falcon, BD Biosciences) were placed into the wells of 24-well culture plates, separating the upper and the lower chambers. In the lower chamber, 600 μL DMEM containing 10% FBS was added. Then 1 × 10^5^/well cells were added to the upper chamber. After 24 h incubation, cells that had migrated through the transwell membrane were stained with crystal violet, and counted (four high power fields, ×100 magnification). In addition, wound healing assay was also performed for analysis of cell migration *in vitro.* Briefly, cells transfected with either miR-139 mimics or and negative control miRNA were cultured in six-well plates (5 × 10^5^ cells per well). When the cells grew up to 90% confluence, a single wound was made in the center of cell monolayer using a P-200 pipette tip. The wound closure areas were visualized under phase-contrast microscope with a magnification × 100, and the migrated cells were counted. The experiment was performed in triplicate wells for three times.

### Cell invasion assay

Cell invasion was performed by Matrigel invasion assay (BD Biosciences). SW1116 and DLD1 cells transfected with pre-miR-139 or control for 48 h were harvested, suspended (1 × 10^5^/well) in 500 uL serum-free medium and then loaded onto the upper compartment of chamber. The lower chamber contains 600 uL DMEM media and 10% FBS. After 24 h incubation, cells that had invaded through the Matrigel membrane were stained with crystal violet, and counted (five high power fields, ×100 magnification). Three independent experiments were conducted.

### Vector construction and dual-luciferase reporter assay

The potential miR-139-5p binding sites were predicted by TargetScan (http://www.targetscan.org) and miRanda (http://www.microRNA.org). Sequence of 42 bp segment with the wild-type or mutant seed region of *NOTCH1* was synthesized and cloned into pMIR-REPORT luciferase vector (Applied Biosystems; Life Technologies). The mutant *NOTCH1* 3’UTR sequence was prepared by deleting 10 nucleotides in the seed region. The synthesized oligos were shown as follows: Wild type of *NOTCH1*: 5’-CTA GTG ACT TTA AAA GTG ATC TAC ATG AGG AAC TGT AGA TGA TGT GAG CT-3’; 5’- CAC ATC ATC TAC AGT TCC TCA TGT AGA TCA CTT TTA AAG TCA-3’; Mutant type of *NOTCH1*: 5’- CTA GTG ACT TTA AAA GTG ATC TAC ATG AGT GAT GTG AGC T-3’; 5’- CAC ATC ACT CAT GTA GAT CAC TTT TAA AGT CA-3’; Cells (1 × 10^5^/well) transiently transfected with pre-miR-139 or control (at 30 nM final concentration) were seeded in 24-well plates. pMIR-REPORT vector (195 ng/well) and pRL-TK vector (5 ng/well) were cotransfected using lipofectamine 2000 (Invitrogen). Cells were harvested 48 hours post-transfection and luciferase activities were analyzed by the dual-luciferase reporter assay system (Promega, Madison, WI).

### Western blot analysis

Total protein was extracted and protein concentration was then measured by the DC protein-assay method of Bradford (Bio-Rad, Hercules, CA). A total of 20 mg protein from each sample was used for western blotting. The primary antibodies for caspase 3, caspase 7, caspase 8, PARP, p27 ^Kip1^ and NOTCH1 were purchased from Cell Signaling Technology (Danvers, MA, USA). Antibodies for p53, p21^Cip1/Waf1^, MMP7, MMP9 and GAPDH from Santa Cruz Biotechnology (Santa Cruz, CA, USA). Proliferating cell nuclear antigen was purchased from Abcam. Bands were quantified by scanning densitometry. All of the western blot data have been repeated three times independently and only representative images were showed in the Figures.

### *In vivo* tumorigenicity

miR-139-5p expression plasmid pCMV-139-5p and control plasmid pCMV-Ctrl (Origene, Rockville, MD) were delivered into cells using Lipofectamine 2000 and stable cell lines were created from puromycin-resistant colonies. HCT116 cells (2 × 10^6^) stably expressing pCMV-139-5p or HCT116 control cells (pCMV-Ctrl) were injected subcutaneously into the left flank of the female BALB/c nude mice (4 weeks old), respectively (n = 4 mice per group). Tumour size was measured every 3 day using caliper, and the tumor volume (*V*) was calculated as (*l* × *w* × *w*)/2, with *l* indicating length and *w* indicating width. Mice were killed by cervical dislocation in day 28, and the tumors were excised and snap-frozen for protein extraction. All experimental procedures were approved by the Animal Ethics Committee of the Chinese University of Hong Kong.

### Statistical analysis

Statistical analysis was carried out using SPSS 16.0 for Windows. All measurements or variables were shown as mean ± SD. Results of miR-139-5p level between paired samples determined by the Wilcoxon matched pairs test. Results of colony formation assays, flow-cytometry analyses, cell growth, migration and invasion assays were analyzed by student *T* test. Repeated Measures ANOVA was used to compare the tumor growth rate between two groups in the *in vivo* assay. Based on the expression levels in tumor, the miR-139-5p expression in CRCs was categorized as high (miR-139-5p level in tumor > =median) and low (miR-139-5p level in tumor < median). The Pearson's chi-squared test was used to analyse the association of miR-139-5p expression and clinical-pathological parameters. Kaplan-Meier tests were used for survival analysis. Overall survival times were calculated from the date of curative surgery to death or last follow-up of patients. A *P* < 0.05 was taken as statistical significance.

## Abbreviations

3’ UTR: 3’ untranslation region; 7-AAD: 7-aminoactinomycin D; APC: Annexin V-Allophycocyanin; ATCC: American type culture collection; CDK: Cyclin dependent kinase; CFLAR: FADD-like apoptosis regulator; CRC: Colorectal cancer; FBS: Fetal bovine serum; GAPDH: Glyceraldehyde 3-phosphate dehydrogenase; HES1: Hairy and enhancer of split-1; IQR: Interquartile range; mRNA: Messenger RNA; miRNA: microRNA; MMP: Matrix metalloproteinases; PARP: Poly-(ADP-ribose) polymerase; PCR: Polymerase chain reaction; qRT-PCR: Quantitative real-time polymerase chain reaction; RNU6B: U6 small nuclear 2.

## Competing interests

The authors declared that they have no competing interests.

## Authors’ contributions

LZ, YD, NZ, HT, KW designed and performed the experiments, analyzed data and wrote the manuscript; CWW, SSMN, SZ, FKLC and JJYS provided technical and material support; ZZ provided manpower and material support, JY supervised the project, analyzed data and revised the manuscript. All authors read and approved the final manuscript.

## Authors’ information

Zengren Zhao Co-corresponding author.

## Supplementary Material

Additional file 1: Table S1 Clinicopathologic Characteristics of 95 patients with colorectal cancer included in the study. Click here for file

Additional file 2: Figure S1 **(A)** The expression level of miR-139-5p was restored in CRC cell lines upon administration of DNA methylation inhibitor 5-aza-2’-deoxycytidine. 5AZA: 5-aza-2’-deoxycytidine. **(B)** Overexpression of Pre-miR-139 increased the miR-139-5p level in HCT116, DLD1 and SW1116. Click here for file

Additional file 3: Table S2 Correlation between miR-139-5p expression and clinicopathologic parameters in 44 CRC patients (cohort 1). Click here for file

Additional file 4: Figure S2 Kaplan-Meier survival curves for patients with colorectal cancer in Cohort 1, stratified according to miR-139-5p **(A and B)** or *NOTCH1***(C and D)** expression levels. The follow-up data for one patient is not available, n = 44. Click here for file

Additional file 5: Figure S3 Overexpression of miR-139-5p in HCT116 significantly suppressed cell migration ability as determined by wound-healing. Click here for file

Additional file 6: Figure S4 Role of NOTCH1 in colon cancer. **(A)** Knock-down of NOTCH1 was evidenced by markedly decrease of NOTCH1 protein expression in HCT116 cells by si-NOTCH1. **(B)** NOTCH1 knock-down significantly suppressed cell viability in HCT116 cells. **(C)** si-NOTCH1 caused cell cycle arrest at G0/G1 phase in HCT116. Quantitative analysis of DNA profile in HCT116 (right panel). The experiments were repeated three times in triplicate. Data are mean ± SD. Click here for file

Additional file 7: Figure S5 miR-139-5p showed no significant effect on JUN in HCT116. **(A)** Human JUN 3’UTR binding site for miR-139-5p. **(B)** The miR-139-5p wild type binding sequence or its mutated form was inserted into C-terminal of the luciferase gene to generate pMIR-JUN-3’UTR or pMIR-JUN-mut-3’UTR, respectively. **(C)** miR-139-5p showed no significant effect on the wild-type or the mutant 3’UTR of JUN. The data are means ± SD. Click here for file

Additional file 8: Table S3 Correlation between *NOTCH1* mRNA expression and clinicopathologic parameters in 44 CRC patients (cohort 1). Click here for file
