# Supplementary material for: microRNA-139-5p exerts tumor suppressor function by targeting NOTCH1 in colorectal cancer
Source: Mol Cancer. 2014 May 26;13:124. doi: 10.1186/1476-4598-13-124 (PMC4065091; doi:10.1186/1476-4598-13-124)
Supplement: Additional file 1: Table S1 — Clinicopathologic Characteristics of 95 patients with colorectal cancer included in the study. [file 1476-4598-13-124-S1.docx]

Additional file 3: Table S2. Correlation between miR-139-5p expression and clinicopathologic parameters in 44 CRC patients (cohort 1).

| **Variables** | **miR-139-5p expression*** | | **Statistics** | ***P* value** |
| --- | --- | --- | --- | --- |
|  | **Low (n =23)** | **High (n =21)** |  |  |
| Age, year | 65.18±12.18 | 67.3±12.22 |  |  |
| Sex |  | | 0.159 | 0.690 |
| Male | 14 | 14 |  |  |
| Female | 9 | 7 |  |  |
| Lymph node metastasis |  |  | 2.187 | 0.139 |
| No | 16 | 10 |  |  |
| Yes | 7 | 11 |  |  |
| TNM stage |  |  | 2.609 | 0.271 |
| I | 5 | 2 |  |  |
| II | 11 | 8 |  |  |
| III | 7 | 11 |  |  |
| Tumor size |  |  |  |  |
| <5 cm | 13 | 16 | 0.287 | 0.592 |
| >=5cm | 8 | 7 |  |  |
| Recurrence |  |  | 2.257 | 0.133 |
| No | 18 | 12 |  |  |
| Yes | 5 | 9 |  |  |
| Outcome |  |  | 2.199 | 0.138 |
| Alive | 17 | 11 |  |  |
| Dead | 6 | 10 |  |  |
| Disease-free survival month Mean( SD) | 73.61 (37.83) | 64.74 (40.72) | -1.187 | 0.235 |
| Total survival Mean( SD) | 83.02 (28.52) | 81.14 (28.81) | -0.317 | 0.751 |

*The follow-up for one patient is not available.
