# Supplementary figures and images for: microRNA-139-5p exerts tumor suppressor function by targeting NOTCH1 in colorectal cancer
Source: Mol Cancer. 2014 May 26;13:124. doi: 10.1186/1476-4598-13-124 (PMC4065091; doi:10.1186/1476-4598-13-124)

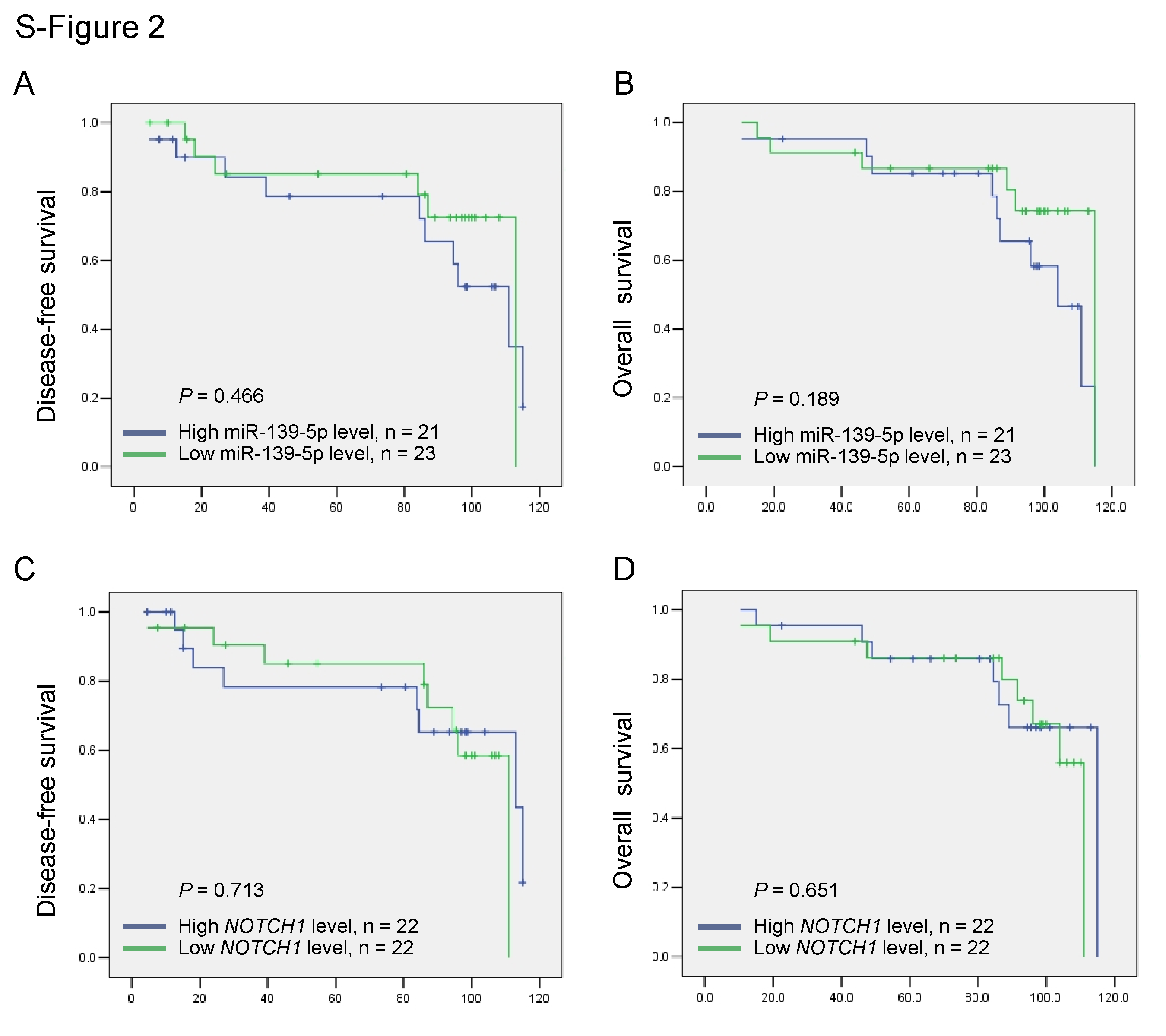

Supplement: Additional file 2: Figure S1 — (A) The expression level of miR-139-5p was restored in CRC cell lines upon administration of DNA methylation inhibitor 5-aza-2’-deoxycytidine. 5AZA: 5-aza-2’-deoxycytidine. (B) Overexpression of Pre-miR-139 increased the miR-139-5p level in HCT116, DLD1 and SW1116. [file 1476-4598-13-124-S2.tiff]

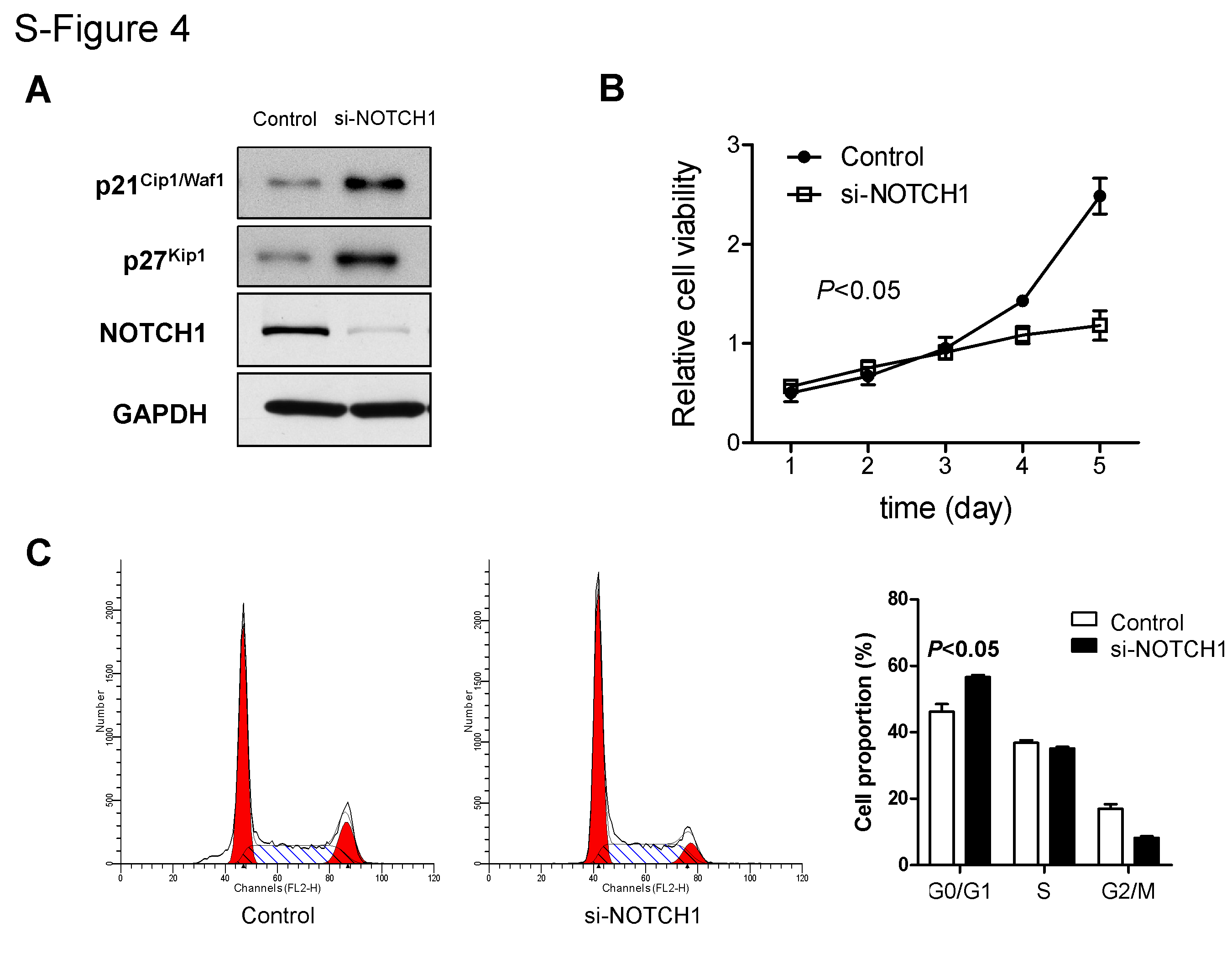

Supplement: Additional file 4: Figure S2 — Kaplan-Meier survival curves for patients with colorectal cancer in Cohort 1, stratified according to miR-139-5p (A and B) or NOTCH1(C and D) expression levels. The follow-up data for one patient is not available, n = 44. [file 1476-4598-13-124-S4.tiff]

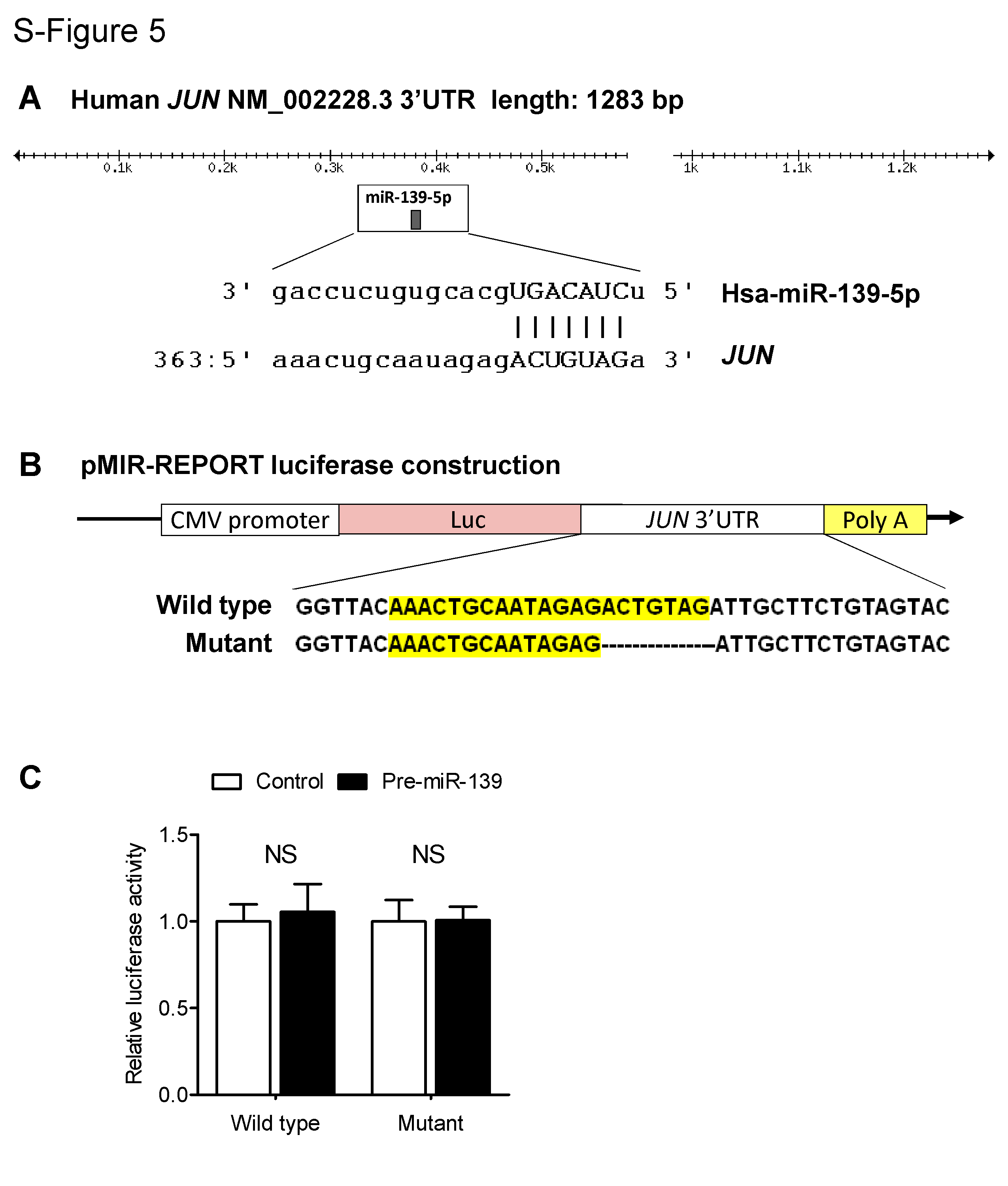

Supplement: Additional file 5: Figure S3 — Overexpression of miR-139-5p in HCT116 significantly suppressed cell migration ability as determined by wound-healing. [file 1476-4598-13-124-S5.tiff]
