# Supplementary material for: microRNA-139-5p exerts tumor suppressor function by targeting NOTCH1 in colorectal cancer
Source: Mol Cancer. 2014 May 26;13:124. doi: 10.1186/1476-4598-13-124 (PMC4065091; doi:10.1186/1476-4598-13-124)
Supplement: Additional file 8: Table S3 — Correlation between NOTCH1 mRNA expression and clinicopathologic parameters in 44 CRC patients (cohort 1). [file 1476-4598-13-124-S8.docx]

Additional file 8: Table S3. Correlation between *NOTCH1* mRNA expression and clinicopathologic parameters in 44 CRC patients (cohort 1).

| **Variables** | **NOTCH1 expression*** | | **Statistics** | ***P* value** |
| --- | --- | --- | --- | --- |
|  | **Low (n =22)** | **High (n =22)** |  |  |
| Age, year | 65.54 (14.08) | 66.14 ( 9.59) |  |  |
| Sex |  | | 0.393 | 0.531 |
| Male | 15 | 13 |  |  |
| Female | 7 | 9 |  |  |
| TNM stage |  |  | 2.371 | 0.306 |
| I | 3 | 4 |  |  |
| II | 12 | 7 |  |  |
| III | 7 | 11 |  |  |
| Lymph node metastasis |  |  | 1.504 | 0.22 |
| No | 15 | 11 |  |  |
| Yes | 7 | 11 |  |  |
| Tumor size |  |  |  |  |
| <5 cm | 11 | 17 | 3.536 | 0.06 |
| >=5cm | 11 | 5 |  |  |
| Recurrence |  |  | 0.419 | 0.517 |
| No | 16 | 14 |  |  |
| Yes | 6 | 8 |  |  |
| Outcome |  |  | 0 | 1 |
| Alive | 14 | 14 |  |  |
| Dead | 8 | 8 |  |  |
| Disease-free survival month Mean( SD) | 72.41 (36.98) | 66.27 (41.73) | -0.622 | 0.534 |
| Total survival Mean( SD) | 83.7 (28.72) | 80.54 (28.55) | -0.563 | 0.573 |
|  |  |  |  |  |

* The follow-up for one patient is not available.
